# A PROgramme of Lifestyle Intervention in Families for Cardiovascular risk reduction (PROLIFIC Study): design and rationale of a family based randomized controlled trial in individuals with family history of premature coronary heart disease

**DOI:** 10.1186/s12889-016-3928-6

**Published:** 2017-01-05

**Authors:** Panniyammakal Jeemon, S. Harikrishnan, G. Sanjay, Sivasankaran Sivasubramonian, T. R. Lekha, Sandosh Padmanabhan, Nikhil Tandon, Dorairaj Prabhakaran

**Affiliations:** 1 Public Health Foundation of India, New Delhi, India; 2 Sree Chitra Tirunal Institute of Medical Sciences and Technology, Trivandrum, India; 3 Institute of Cardiovascular and Medical Sciences, University of Glasgow, Glasgow, UK; 4 All India Institute of Medical Sciences, New Delhi, India

## Abstract

**Background:**

Recognizing patterns of coronary heart disease (CHD) risk in families helps to identify and target individuals who may have the most to gain from preventive interventions. The overall goal of the study is to test the effectiveness and sustainability of an integrated care model for managing cardiovascular risk in high risk families. The proposed care model targets the structural and environmental conditions that predispose high risk families to development of CHD through the following interventions: 1) screening for cardiovascular risk factors, 2) providing lifestyle interventions 3) providing a framework for linkage to appropriate primary health care facility, and 4) active follow-up of intervention adherence.

**Methods:**

Initially, a formative qualitative research component will gather information on understanding of diseases, barriers to care, specific components of the intervention package and feedback on the intervention. Then a cluster randomized controlled trial involving 740 families comprising 1480 participants will be conducted to determine whether the package of interventions (integrated care model) is effective in reducing or preventing the progression of CHD risk factors and risk factor clustering in families. The sustainability and scalability of this intervention will be assessed through economic (cost-effectiveness analyses) and qualitative evaluation (process outcomes) to estimate value and acceptability. Scalability is informed by cost-effectiveness and acceptability of the integrated cardiovascular risk reduction approach.

**Discussion:**

Knowledge generated from this trial has the potential to significantly affect new programmatic policy and clinical guidelines that will lead to improvements in cardiovascular health in India.

**Trial registration number:**

NCT02771873, registered in May 2016 (https://clinicaltrials.gov/ct2/show/results/NCT02771873)

## Background

Coronary heart disease (CHD), now the leading cause of death and disability [1], strikes mostly in the productive mid-life years and at least a decade earlier in Indians in comparison to their Caucasian counterparts [2–4]. A positive family history (FH+) is well-recognised as a consistent and independent risk factor for CHD [5] featuring in several cardiovascular risk prediction scores commonly used in clinical practice [6–9]. Additionally, the FH+ of premature vascular disease was associated with a greater lifetime risk of CHD mortality in a large cohort of men (*n* = 49,255) with low risk factor burden [10]. More importantly, the increased risk of cardiovascular disease conferred by FH+ is mediated in large part by modifiable risk factors and thus family history is helpful in identifying individuals who may have the most to gain from preventive interventions.

While FH+ can influence greater awareness of risk with early and possibly more intensive risk factor modification measures, it is unclear if these measures lead to reversal of the increased risk associated with these individuals. Current preventive management of individuals with FH+ of CHD is however ‘reactive’ based on development of risk factors rather than ‘proactive’ global risk reduction measures of reducing total cardiovascular risk. In a recent study, treated hypertensive individuals with a FH+ of CHD reported better longitudinal reduction in blood pressure (BP) and this is consistent with the recommendations of intensive management of risk factors in individuals with FH+ [11].

Lifestyle changes are likely to be more effective when delivered to the whole family than to individuals as it works within the framework of biologic and cultural relationships to affect risk reduction. Family-based approaches that target the whole family, encourage communication among the family unit, and address the structural and environmental conditions in which families live and operate, are considered to be effective approaches to promote cardiovascular health [12]. Although cardiovascular risk factors cluster together in individuals, comprehensive interventions involving family members reduced the magnitude of risk factor clustering in a worksite based demonstration project in India [13, 14]. However, how cardiovascular interventions can be developed in a culturally appropriate manner in family settings has not been explored in detail in a systematic way. We propose to use mixed methods (qualitative research, randomized control trial (RCT), and cost-effectiveness analysis) to integrate cardiovascular risk reduction strategies in high risk families with FH+ of premature CHD and evaluate their effectiveness.

## Methods

The major aims of the study are as follows: (1) to identify barriers to implementing an integrated cardiovascular risk management program in families of individuals with a positive history of CHD, (2) to assess the effectiveness of an integrated cardiovascular risk management strategy (consisting of screening for risk factors, lifestyle education and linkage to primary care for cardiovascular risk factor management) on modifying risk factor clustering in families, and to assess changes in BP, lipids, glucose, haemoglobin A1c (HbA1c), smoking, diet and physical activity and (3) to estimate the scalability of the integrated cardiovascular risk reduction strategy in families of individuals with a positive history CHD for state- or nation-wide implementation.

The proposed project uses mixed methods to achieve the study aims. The three design approaches include: Aim 1, formative qualitative research; Aim 2, a randomized controlled trial; and Aim 3, cost effectiveness and evaluative qualitative research to inform acceptability and scalability.

### Formative qualitative research (Aim 1)

Semi-structured focus group discussions (FGDs) of 6–12 adults with FH+ of CHD will gather information on understanding of diseases, barriers to care, and feedback on the intervention (lifestyle education, care and linkage coordination) (Table 1). Similarly, FGDs will be conducted among other stakeholders such as frontline community health workers (FCHW). Key informant interviews (*n* = 6) will also be conducted among primary care physicians. The qualitative approach will largely focus on recognising solutions to previously identified potential barriers that may impede first-degree relatives from engaging in risk-reducing behaviors [15] and they include: (1) failure to understand the importance of family history as a risk factor for heart disease; (2) denial of a FH+, inappropriate risk perception or underestimation of one’s own vulnerability; (3) failure of health workers to screen and provide advice; and (4) inability to sustain risk-reducing behaviors after implementation and poor control among those in treatment. The intervention and trial protocols will be then modified to incorporate key findings collected from focus groups to make the intervention more contextually-relevant.

**Table 1:** Formative qualitative methods to inform cardiovascular risk management integration

Method	Participants	Number	Topics
Focus Groups	Individuals with family history of AMI -Males groups aged 18-40, 40+ -Female groups aged 18-40, 40+	-3 discussions in each gender-age category, (12 total discussions) -Each focus groups include 6-12 participants	• Family history as a risk factor for future cardiovascular event. • Perceptions and behaviours regarding other risk factors of CHD • Suggested intervention components and methods • Gauge community interest in planned intervention components
	Frontline community health workers	-4 focus groups -Each focus groups include 6-12 participants	• Perceived patient barriers to lifestyle change, and an integrated cardiovascular risk reduction initiative • Gauge feasibility of planned intervention components

### Integrated cardiovascular risk reduction intervention (Aim 2)

**Fig. 1:**
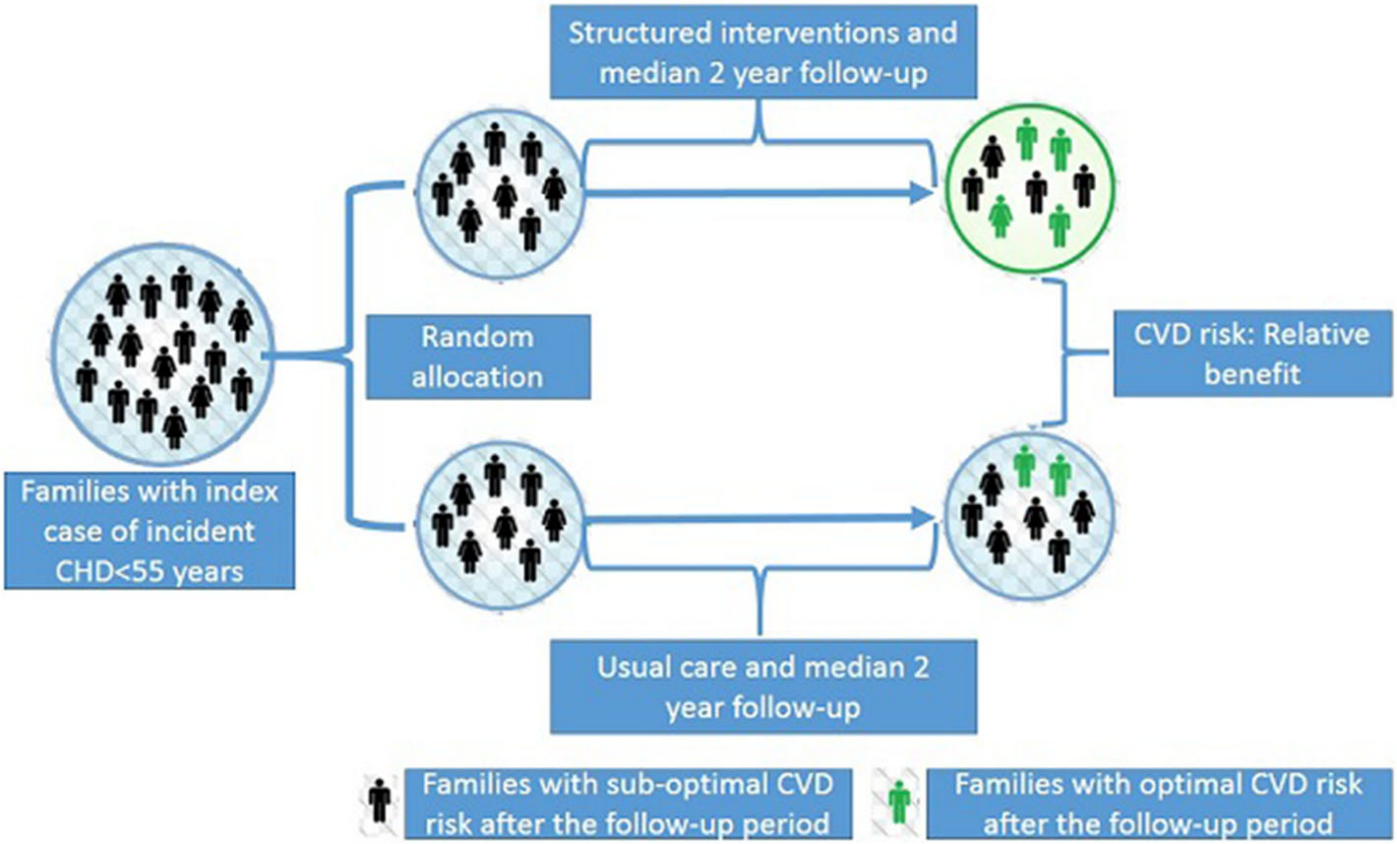
Schematic diagram of the randomized controlled trial. CHD = Coronary heart disease, CVD = Cardiovascular disease

**Fig. 2:**
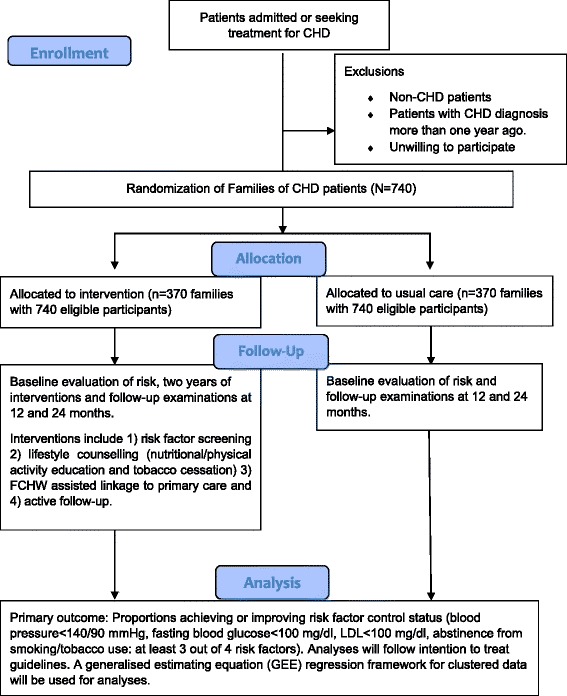
CONSORT flow diagram of the randomized controlled trial design. CHD = Coronary heart disease, FCHW = Frontline community health worker

A randomized controlled trial of families of individuals with a positive history of CHD will be used to address Aim 2 (Fig. 1). *Eligibility criteria*: Eligible families will include those with at least one family member with physician confirmed CHD diagnosed before the age of 55 years. If there are family members who have died from CHD (physician confirmed deaths) before the age of 55 years, such families will also be eligible to participate in the study. The medical records of CHD patients will be used to confirm the diagnosis and the age of the index case. A written informed consent to the study randomization plan from the head of the family will be a mandatory eligibility criterion. All participating family members will be required to provide individual consent to participate. *Family randomization*: Each family will be randomly assigned to participate in either the treatment intervention arm (integrated CVD risk management) or usual care arm (no interventions other than initial screening). Computer generated random numbers generated by an independent member who is not involved in the study will be used for randomization. Randomization of a family will be performed only after collection of complete baseline data. The outcome assessors and the data manager will be blinded from the intervention assignment. The data analysts will be provided with the intervention assignment only after complete database lock. *Participant eligibility criteria*: A minimum of two members from each family will be included even if they reside in separate houses. Potential participants must be more than 18 years of age and either first degree blood relatives or spouses of the index case. Bedridden and terminally ill patients will be excluded from the study. The detailed study flow chart is presented in Fig. 1. *Follow-up period:* All participants will be followed-up for two years after randomization. Study related measurements will be conducted at baseline, one year and two year time-points. The full study flow chart is presented in Fig. 2.

#### Data collection

A structured questionnaire will be administered by trained staff to collect relevant data at baseline, one year and two year time-points. The questionnaire includes assessments of demographic and socio-economic variables, general health status, diet pattern, physical activity, tobacco, and alcohol consumption. The World Health Organization (WHO) STEPS instrument for chronic risk factor surveillance has been modified and adapted to capture local, contextual information [16]. Dietary data will be collected using a semi-quantitative food frequency questionnaire on food items that are relevant to chronic diseases and adapted from an ongoing cluster randomized trial in India [17]. Additionally, all participants will undergo anthropometric and BP measurements at three time points (baseline, year 1 and year 2). Anthropometric measurements include height (in cm), weight (in kg) and waist circumference (in Inches). BP and pulse rate will be measured using electronic BP monitors (OMRON HEM-7130-L) at all three time-points. Three measurements will be taken at the respective houses of the participants by trained staff, two minutes apart and after allowing the participant to relax for at least five minutes before starting the measurement. Participants will be instructed not to consume any beverages (coffee, tea or soft drinks) and alcohol at least one hour before taking the measurements. They will also be instructed to abstain from smoking. In fasting stage, 5 ml of blood will be collected at three time-points, centrifuged at source for serum and plasma separation, and transported to the study laboratory. They will be stored under −20 °C freezer. The following biochemical analyses will be performed on the collected blood samples: lipid profile (total cholesterol, LDL cholesterol, HDL cholesterol and VLDL cholesterol), fasting plasma glucose, and haemoglobin A1c (Table 2). Abstinence from tobacco at year 1 and year 2 will be validated by assessing urinary cotinine metabolites. Spot urine samples are collected at year 1 and year 2 for this purpose. The study laboratory is accredited by the National Accreditation Board for testing and calibration of Laboratories (NABL), India and also part of international external quality assurance programs (EQAS) such as EQAS of BIO-RAD.

**Table 2:** Study measurements

Study parameters	Method/Instrument
Blood pressure in mmHg	Electronic BP monitor (OMRON HEM-7130-L). Instrument validated by International Protocol for device validation O’Brien et al, (Working Group on Blood Pressure Monitoring of the European Society of Hypertension)
Height in Cm	Stadiometer (Seca 213)
Weight in meter	Digital weighing scales (Seca HN286)
Wait circumference in inches	Non elastic measuring tapes (Seca 201)
Plasma glucose in mg/dl	Enzymatic Colorimetric Assay method (modified GOD-PAP method based on the work of Trinder, 1969)
Glycosylate Hb (HbA1c), %	High Performance Liquid Chromatography (HPLC)
Cholesterol in mg/dl	CHOD-PAP enzymatic. Enzymatic In vitro Calorimetric method (automated clinical chemistry analyzer Roche/Hitachi 902)
Triglycerides in mg/dl	Enzymatic Calorimetric test, GPO-PAP method
High density lipoprotein cholesterol (HDL) in mg/dl	Direct measurement. Automated method for direct determination.
Low density lipoprotein cholesterol (LDL)	Direct measurement. Homogeneous Enzymatic Assay for direct quantitative determination (automated clinical chemistry analyzer Roche/Hitachi 902), for samples with triglycerides more than 400 mg/dl.
Very low density lipoprotein cholesterol (VLDL)	Estimation using Friedewald and Fredrickson Formula, 1972
Nicotine metabolites screening in urine.	Lateral flow chromatographic immunoassay for the detection of cotinine in human urine at the cut-off concentration of 200 ng/ml.

*mg/dl* milligram/deci litre, *Cm* Centimetre, *mmHg* milli meters of mercury, *nd ng/dl* nano gram/deci litre

#### Study outcomes

To address Aim 2, several clinical outcomes are assessed. *Primary outcome*: proportions in each group achieving or improving risk factor control status (BP < 140/90 mmHg, fasting blood glucose (FPG) < 110 mg/dl, low-density lipoprotein (LDL) < 100 mg/dl, abstinence from smoking/tobacco use: at least 3 out of 4 risk factors). Secondary outcomes: 1) between group difference in number of optimal cardiovascular health indicators (BP < 120/80 mmHg, TC < 200 mg/dl, FPG < 100 mg/dl, >150 min/week of moderate intensity physical activity, BMI < 25 kg/m2, never used tobacco or quit >6 months ago and a healthy diet score) [18], 2) between group mean difference in main risk factors (SBP, DBP, LDL, FPG, and HbA1c), 3) proportions meeting >80% of recommended process measures (smoking cessation, moderate or high intensity physical activity, and <5 g per person/day salt consumption, >3 daily servings of fruits and vegetables, <2 table spoons/cubes of free sugar), and 4) proportions achieving or maintaining ten year Framingham cardiovascular disease (CVD) risk score [19]/WHO CVD risk score [20] of <10% or the INTERHEART non-laboratory based score of <5 [21].

### Study intervention overview

Usual care arm: The usual care arm will be screened for CVD risk factors. Screening results plus one time education regarding management of risk factors will be provided to all family members. Patients with hypertension, diabetes and dyslipidemia in the control arm will be also referred to a primary health care facility. Treatment arm: The study intervention is designed to leverage the existing health care infrastructure available at the community level. Trained FCHW will be visiting the families to promote lifestyle intervention strategies at least once in two months during the intervention phase. The intervention will consist of three phases: 1) screening and detection of CVD risk factors, 2) lifestyle (nutritional/physical activity education and tobacco cessation) counselling 3) FCHW assisted linkage to primary care and 4) active follow-up through a mobile phone application.

#### Intervention Part 1. Screening for CVD risk factors (CONTROL AND TREATMENT ARMS)

All eligible adults in the selected families who provide written informed consent will undergo a screening procedure for CVD risk factors. The families will then be randomized to either the treatment or control arm. At the start of the intervention program, high risk patients in the usual care arm will be referred to the existing primary care system for further management.

#### Intervention Part 2. Lifestyle Education (TREATMENT ARM)

Lifestyle education intervention: The lifestyle education component of the treatment arm will include 9–12 nutritional/tobacco/physical activity consultations. All study participants within the selected families in the treatment arm will participate in the lifestyle education component. Within 30 days of study enrolment, all participants will complete culturally specific semi-quantitative food frequency questionnaires (FFQ) and tobacco use surveys. FCHW will receive training from study staff prior to study implementation. Recommended diet modifications for participants will focus on maintaining a healthy weight via redistribution/reduction of calories (by avoiding fried foods and sugar-sweetened beverages), increasing fiber and protein intake via wheat and sprouted pulses, reducing glycaemic load by switching from refined white rice and bread to whole wheat, increasing fruit and vegetable consumption, and reducing salt and sugar intake. Tobacco cessation and strategies to change sedentary behaviour will also be discussed in the nutritional/tobacco/physical activity consultations. Current tobacco users will complete culturally appropriate cessation action guides that identify triggers and action steps to promote tobacco-free behaviours. Strategies to improve physical activity levels of all members of the family will be discussed at least two times during the intervention phase. Family consultations will be repeated every two months to assess dietary, physical activity and tobacco use changes. FCHW workers will also conduct peer-support group education meetings with groups of 10 participants/families once in two months. Support group meetings will review diet modification and tobacco cessation recommendations and highlight peers’ strategies that have led to successful improvements in diet. All individuals with elevated CVD risk factors will be invited to participate in at least 3 such peer-led support groups during the period of the trial.

Linkage to primary care system: All high risk patients from the treatment arm will be linked to appropriate primary care clinics with assistance from FCHWs. FCHWs will assist high risk patients to identify primary or secondary care facilities and help them schedule initial appointments. For 24 consecutive months following initial screening and randomization, participants from the intervention arm will receive quarterly phone interviews to measure primary care clinic utilization and to assess CVD risk factor management activities (lipid examinations, smoking cessation programs, hypertension control, glucose monitoring).

#### Materials for intervention

We will adapt previously developed strategies in similar studies for development of the intervention materials [17]. Initially, each component of the healthy lifestyle behaviour related to CVD risk reduction (diet, physical activity, tobacco, alcohol and adherence to treatment) will be broken down into performance objectives for the family. Given the practical feasibility of implementation, resource constraints and the local context, these components will be prioritized. In the final step, key potential change objectives and the corresponding intervention options will be set at both the individual and family level. To communicate the selected change objectives clearly and concisely, we will develop different tools after conducting several FGDs with the potential participants and FCHWs. Based on their inputs, materials such as pamphlets for the family, a hanging calendar to describe the dietary priorities for cardiovascular health in the family and an individual diary to set behavioural goals for each month will be developed. These materials will undergo translation into the local language and will be used during FCHW training. They will ultimately serve as communication aids between the FCHW and family members.

#### Care coordination and follow-up

Each family in the treatment arm will be assigned a FCHW. During the intervention phase, FCHW workers will meet with study patients once in two months to assess study progress and participation. In addition, questionnaire data collection and blood draws at home visits after study months 12 and 24 will help to assess detailed attributes of cardiovascular management (FPG, HbA1c, BP, and LDL measures). A mobile phone SMS platform will be used to enhance follow-up. Via SMS, patients will receive study visit reminders and prompts to encourage adherence to treatment and lifestyle goals.

### Cost effectiveness and acceptability analyses (Aim 3)

The intervention costs will include the resource inputs (e.g., personnel salaries, and materials) that are required to deliver the intervention (in total and per participant). Direct and indirect costs incurred by each family will be estimated using questionnaires administered at study visits 0, 12 and 24. Questionnaires will capture direct medical costs, direct non-medical costs (travel), and indirect costs (missed work time, and lost productivity). To estimate the cost-effectiveness (cost per one count decrease in clustering of risk factors), the between trial group difference in intervention delivery and patient direct costs will be divided by the between group difference in clustering of risk factors or cardiovascular risk score: [costs treatment arm – costs standard arm]/[ΣΔ Primary outcome-Treatment arm] – [ΣΔ Primary outcome-usual care arm]. Similarly, incremental cost utility ratio will also be calculated using data from a 12 monthly health utility assessment. Quality adjusted life years (QALY) will be used as the measure of health utility. The acceptability of the interventions will be assessed through qualitative research. Semi-structured FGDs (4-6 each in men and women) and in-depth interviews with FCHW (6 interviews) will be used to gauge the acceptability of the interventions.

### Trial sample size and power

The sample size estimates accounts for the expected dependency of variables [22]. This family based RCT with 740 families (370 in each arm) of at least two participants each (minimum of 1480 enrolled participants) and no more than 15% loss to follow-up after 2 years will have 90% power to detect an estimated 10% difference in the proportion of participants at 3 or more CHD risk factor goals, the primary effectiveness endpoint of this trial (55% in the intervention group as compared to the expected rate of 45% in the control group). The baseline rate of the composite primary outcome in a large cluster randomized trial in India [17] is used as the expected rate in the control group (data not published) and the lower limit of 95% confidence interval of the improvement in the same composite outcome in a demonstration project in India [13] is used to estimate the rate in the intervention group (data not published). This is for a two sided test with an alpha of 0.05 and a rho or intra-cluster correlation coefficient (ICC) of 0.20. The ICC of the composite primary outcome is also estimated from the baseline data of the above mentioned cluster randomized trial [17]. The power in this study is more than 80% for all secondary endpoints of interest including the change in risk scores.

### Participating centres

The study will be conducted in Kerala with active support from Sree Chitra Tirunal Institute of Medical Sciences and Technology (SCTIMST) in Trivandrum in India. In total, 3716 patients with premature (<55 years) non-fatal acute coronary syndrome were registered in this hospital in the last ten years. The average number of cases per year in this center is in the range of 300–500. Nearly 500 patients are registered in the last one year alone. We will invite 740 such families in this trial and the recruitment will involve both prospective cases as well as cases in the last 1 year. The medical records will be used to confirm the diagnosis of CHD and the eligibility criteria (Age < 55 years).

### Data entry and data management

Trained and certified project staff will collect data using study questionnaires. They will also be trained in collection of clinical data from health records and/or personal information. The project staff under the supervision of a post-doctoral fellow and the principal investigator will complete data entry on a specially designed tablet computer based data application. The data application has been developed on My SQL 7.0 server. The application will upload the data directly on a cloud server. The data will be collated, cleaned, queries resolved and analysed centrally. All entries in the tablet application will be double checked by the post-doctoral fellow using a specially designed data review application. The application also allows the user to send queries directly back to the field staff for their review and action. Further, the accuracy of data entry in randomly selected fields (10% of the general fields and all anthropometric, BP, and biochemistry data) will be checked independently by the study principal investigator.

The study will collect various type of research data including both quantitative and qualitative data. Data will be generated from focus groups, in-depth semi-structured interviews, medical records, questionnaires, clinical and anthropometric measurements and blood biochemistry. All data will be de-identified to ensure confidentiality of information.

Long-term data storage: Upon completion of data collection and entry, data packets will be created containing the following: a final STATA data file; a read me file describing data collection, entry, auditing, and cleaning; a data dictionary with detailed description of study variables, and an annotated PDF of questionnaires and laboratory data with variables and coding noted. De-identified study data will be maintained indefinitely at the secured data servers of the study sponsor. Periodically, the data will be checked to ensure long-term integrity of the datasets. A master list linking study identifiers with individuals will be maintained for five years to allow the researchers to reach individuals if needed to inform them of individual study results that could impact their health. After five years the linkage file will be destroyed. Three years after the completion of the study, study data (de-identified quantitative data including survey and data from clinical records and qualitative data comprising de-identified transcripts) for all participants who consent to having their data made public will be made available to other researchers. Information on the study will be available on the website of the sponsor as well as reported in scientific journals and at scientific conferences. In addition, the study results will be included in clinicaltrials.gov. Interested collaborators can contact the study PI to discuss the possibility of data usage.

### Data analysis plan

All quantitative analyses (for Aim 2) will follow recommended intention to treat guidelines for RCT. A generalizing estimating equations (GEE) framework for clustered data [23] will be used to assess the statistical significance of any observed intervention effect on the primary endpoint. In this analysis, the dependent variable will be the indicator for whether the study participant is at three or more CVD risk factor reduction goals at the end of 12 and 24 months follow-up period, minus the observed probability of this endpoint in the absence of an intervention (rate in control group). An exchangeable working correlation matrix will be used to incorporate the clustering within the families, with a log link and binomial variance specified. The exponentiated point and interval estimates from the intercept of this model will quantify the intervention effect. A similar GEE approach will be used to estimate and test the significance of intervention effects on secondary endpoints. For continuous markers, such as SBP, FPG, HbA1c and LDL, the within-participant difference will serve as the dependent variable in a GEE model as above, with the identity link function and the distribution specified as normal. To assess the causal effect of the intervention adjusted for bias due to non-adherence and loss to follow up, marginal structural models will be fit, embedded around the analysis approaches outlined above [24].

All qualitative data, including that collected for Aim 1 and Aim 3, will comprise the verbatim transcripts of the audio recordings from all focus group discussions and interviews. Audiotapes of focus group discussions/interviews will be transcribed, de-identified, and the transcriptions audited for accuracy. Using ATLAS.ti software to manage the data, analyses of the textual data will follow the thematic analysis (e.g., for suggestions from patients and care givers for the intervention). Key themes will be identified from the data (inductively) and from the discussion guides (deductively), and these themes will be compared using structured comparisons to identify specific issues relevant to sub-groups of participants.

To compare the cost-utility of the intervention to standard arm management, we propose to calculate an incremental cost/utility ratio [net costs to net utility: costs of intervention – costs of control/utility of intervention – utility of control]. To calculate utility, we will use 12-monthly health utility. The chosen measure of utility will be the closest option to a global measure, the quality adjusted life year (QALY], and is calculated as the sum of mean survival time [life years] x utility scores at 12 and 24 months [25]. Both 12 and 24-month costs per QALY will be reported. If the intervention is successful, the modeled long-term cost-utility of the intervention and usual care will be compared using Markov Chain Monte Carlo estimation techniques, controlling for age, gender, and risk factor prevalence under different plausible scenarios for how these may evolve over time [26]. Then it will be compared to reference points from the literature – e.g., ceiling ratios for costs per QALY that are less than three times gross domestic product (GDP) per capita [27, 28] that are considered cost-effective [29] (India’s 2013 GDP per capita was $3990; threshold: ≤$11,100 per QALY).

### Ethical oversight

The participants will be informed about the study and provided with a detailed information sheet. Trained study staff appointed by the principal investigator will obtain written informed consent from all study participants and also from the head of the family. The study is approved by the institutional review boards of the Public Health Foundation of India and Sree Chitra Tirunal Institute for Medical Sciences and Technology. The study protocol is registered with the clinical trial registry clinicaltrials.gov (NCT02771873). All changes in the trial protocol will be informed to the institutional review boards.

## Discussion

A positive FH+ of CHD increases the propensity to develop future cardiovascular events. Targeted CVD prevention activities is therefore an attractive strategy to delay or prevent CVD onset in a relatively high risk and easy to identify population of individuals with FH+ of CHD. Involving family in cardiovascular health promotion, engaging FCHW as agents of change in imparting lifestyle education and counselling at the family level, and harnessing the potential of existing primary care infrastructure for intermediate risk management are the novel strategies of the proposed trial. The trial is also unique in many other ways: (a) the unit of randomization is at the family level, (b) the delivery of lifestyle interventions is at the family level, and finally (c) the study incorporates several existing theories of family process and functions in the intervention development.

Our trial is dependent on the mutual interdependence of the family system. Although the family is viewed as a complex social system, the elements are interconnected, and it is often viewed as a whole. The whole system together frequently interacts with the environment. This is the basic tenet of the family systems theory [30]. The trial also draws strengths from the postulate that a change in an individual’s role can initiate a change in other family members. A life-threatening event in one of the family members in the form of CHD therefore acts as reason for change in health behaviours in others if the change is supported by the family as a whole system with additional support from the external environment (society). In the double ABCX model of family systems [31], the event (A), the family’s resources (B), and the family’s perception of the event (C) all play a part in determining the family’s response to an emerging priority or need (X). The FCHW in our trial utilises this opportunity and positively interacts with the family to make healthy changes not only for the index case but also for other members in the family. The Circumplex model [32] suggests that family cohesion and communication are also important for the development and sustenance of healthy behaviors. We therefore give importance to family counselling by FCHW in our trial to overcome the challenge of poor communication within families to achieve the set goals.

Diet, physical activity and tobacco are the three main areas that we are targeting for changes within the family. Family eating habits largely depend upon food availability and family food supply. Contextually relevant previous studies elaborate household dynamics of food decision-making process and highlight the role of women, children and cost considerations in arriving at a decision [33]. In our study we therefore introduce healthier alternatives of the existing common recipes, without actually changing the food items being prepared, to the homemakers and women in the family. The FCHW discuss these options with them during their routine visits to the family. They also involve children in the family and make them aware of the importance of healthy diet. In our initial qualitative work, we find that the time spent on cooking has declined over time in most of the families due to increased use of moderately processed food or food brought from outside restaurants. Our strategy is also to discourage the use of processed food and food brought from outside as they are generally high in fat, sugar, and sodium.

We also take into account the cultural contexts within the study settings. Snacking and hot beverages (tea/coffee) use are almost universal in most of the families in these settings. We provide suggestions for healthier alternatives for family snacking and encourage family members to reduce the number of cubes/tea spoons of free sugar added to their beverages or snacking items. Similarly, we instruct them to quantify the use of salt, oil and sugar in cooking and to take a cautious effort in reducing the overall quantity of each of these items used within the family. Additionally, culturally relevant options to improve the overall physical activity levels of all members of the family are discussed during the home visits by the FCHW. All discussions are aided by simple and easy to read hand-outs with pictorial representation of the key messages to be conveyed to the family. Specially designed individual diaries are given to all participants in the intervention families to set monthly goals of desirable lifestyle changes. They are also used for tracking individual changes in behaviours on a monthly basis. The FCHW review these diaries once every two months and give feedback to the participants.

In order to make our intervention approach more comprehensive, we have introduced peer support groups for risk reduction in individuals with established risk factors such as diabetes and hypertension or those who are tobacco users. FCHWs organize these peer sessions with the objective to aid behavioural changes that requires support from outside the family.

The FCHWs in our study are trained to facilitate the creation of a family environment that is conducive to cardiovascular health. They are entrusted with the tasks of modifying the family environment to promote the adoption and sustenance of healthy behaviors, modeling appropriate life-style behaviors, and encouraging self-efficacy of the family to adopt and sustain new strategies for overall improvement in cardiovascular health. The FCHWs in India are known by many names such as community health workers (CHW), community health representatives (CHR), anganwadi workers (AWW), junior public health nurses (JPHN), health visitors, health inspectors, and accredited social health activists (ASHA). We largely involve ASHA workers in our study. Traditionally, FCHWs are involved in activities related to maternal and child health and infectious disease management. We train them as community agents to improve cardiovascular health in families. In the era of a rapidly progressing epidemiological transition in India, task shifting/task sharing strategies such as involving FCHWs in non-communicable disease prevention and control are practical, feasible and scalable strategies. Such strategies have been demonstrated to be cost-effective in low and middle income settings [34]. Our study will also provide evidence to support the task shifting/task sharing strategy of involving FCHWs in prevention and control of cardiovascular disease. Even though the family has always been central to effective intervention by FCHWs, their role in nurturing a family in the perspective of total cardiovascular health is new to the field.

Despite improvements in individual risk factors in multiple risk factor intervention trials, its effectiveness on combined cardiovascular events and mortality are yet to be established [35]. In order to establish the effect of these interventions on cardiovascular morbidity and mortality, large, multi-centric strategic trials are necessary. Given that the power of such studies largely depend on the number of outcome events observed during the study period, they often require a longer follow-up period. Our present study with the median follow-up of just two years is not powered to detect changes in CVD morbidity and mortality. Family based approaches that target both the structural and environmental conditions in which a family lives, and address possible barriers and facilitators of adopting a healthy lifestyle need to be also evaluated for their effectiveness to reduce cardiovascular events and mortality in future.

## Implications

The role of intensive lifestyle management at the family level along with linkage to appropriate primary care for cardiovascular risk management have not been explored before. Knowledge generated from this trial has the potential to significantly affect new programmatic policy and clinical guidelines that will lead to improvements in cardiovascular health in India and other low and middle-income countries. The proposed trial will provide key results that address both feasibility and intervention effect of integrated care. The implementable results will address a disease that causes great morbidity and mortality in low- and middle-income countries. From a clinical perspective, little is known about the natural history of cardiovascular risk factors in individuals with positive FH+ of premature CHD. This trial will importantly document the trajectories of BP, blood glucose and blood lipids and determine if the existing primary care infrastructure can be harnessed to improve cardiovascular risk management.

## Trial sponsor

Public Health Foundation of India, Sector 44, Plot 47, Gurgaon, Haryana, India.

## Abbreviations

AIIMS: All India Institute of Medical Sciences
BMI: Body Mass Index
BP: Blood pressure
CHD: Coronary heart disease
CVD: Cardiovascular disease
DBP: Diastolic blood pressure
EQAS: External quality assurance programs
FCHW: Frontline community health workers
FFQ: Food frequency questionnaire
FGDs: Focus group discussions
FH+: Positive family history
FPG: Fasting blood glucose
GDP: Gross domestic product
GEE: Generalised estimating equations
HbA1c: Heamoglobin A1c
ICC: Intra-class correlation coefficient
LDL: Low density lipoprotein
NABL: National Accreditation Board for testing and calibration of Laboratories
PHFI: Public Health Foundation of India
QALY: Quality adjusted life years
RCT: Randomized controlled trial
SBP: Systolic blood pressure
SCTIMST: Sree Chitra Tirunal Instutute for Medical Sciences and Technology
TC: Total cholesterol
WHO: World Health Organization

## References

[1] Mortality GBD, Causes of Death C (2015). Global, regional, and national age-sex specific all-cause and cause-specific mortality for 240 causes of death, 1990-2013: a systematic analysis for the Global Burden of Disease Study 2013. Lancet.

[2] Joshi P, Islam S, Pais P, Reddy S, Dorairaj P, Kazmi K, Pandey MR, Haque S, Mendis S, Rangarajan S (2007). Risk factors for early myocardial infarction in South Asians compared with individuals in other countries. JAMA.

[3] Prabhakaran D, Jeemon P, Roy A (2016). Cardiovascular diseases in India: current epidemiology and future directions. Circulation.

[4] Xavier D, Pais P, Devereaux PJ, Xie C, Prabhakaran D, Reddy KS, Gupta R, Joshi P, Kerkar P, Thanikachalam S (2008). Treatment and outcomes of acute coronary syndromes in India (CREATE): a prospective analysis of registry data. Lancet.

[5] Prabhakaran D, Jeemon P (2012). Should your family history of coronary heart disease scare you?. Mt Sinai J Med.

[6] Hippisley-Cox J, Coupland C, Vinogradova Y, Robson J, May M, Brindle P (2007). Derivation and validation of QRISK, a new cardiovascular disease risk score for the United Kingdom: prospective open cohort study. BMJ.

[7] Ridker PM, Buring JE, Rifai N, Cook NR (2007). Development and validation of improved algorithms for the assessment of global cardiovascular risk in women: the Reynolds Risk Score. JAMA.

[8] Ridker PM, Paynter NP, Rifai N, Gaziano JM, Cook NR (2008). C-reactive protein and parental history improve global cardiovascular risk prediction: the Reynolds Risk Score for men. Circulation.

[9] Woodward M, Brindle P, Tunstall-Pedoe H, estimation Sgor (2007). Adding social deprivation and family history to cardiovascular risk assessment: the ASSIGN score from the Scottish Heart Health Extended Cohort (SHHEC). Heart.

[10] Bachmann JM, Willis BL, Ayers CR, Khera A, Berry JD (2012). Association between family history and coronary heart disease death across long-term follow-up in men: the Cooper Center Longitudinal Study. Circulation.

[11] Williamson C, Jeemon P, Hastie CE, McCallum L, Muir S, Dawson J, Walters M, Sloan W, Morrison D, Dominiczak AF (2014). Family history of premature cardiovascular disease: blood pressure control and long-term mortality outcomes in hypertensive patients. Eur Heart J.

[12] Vedanthan R, Bansilal S, Soto AV, Kovacic JC, Latina J, Jaslow R, Santana M, Gorga E, Kasarskis A, Hajjar R (2016). Family-based approaches to cardiovascular health promotion. J Am Coll Cardiol.

[13] Jeemon P, Prabhakaran D, Goenka S, Ramakrishnan L, Padmanabhan S, Huffman M, Joshi P, Sivasankaran S, Mohan BV, Ahmed F (2012). Impact of comprehensive cardiovascular risk reduction programme on risk factor clustering associated with elevated blood pressure in an Indian industrial population. Indian J Med Res.

[14] Prabhakaran D, Jeemon P, Goenka S, Lakshmy R, Thankappan KR, Ahmed F, Joshi PP, Mohan BV, Meera R, Das MS (2009). Impact of a worksite intervention program on cardiovascular risk factors: a demonstration project in an Indian industrial population. J Am Coll Cardiol.

[15] Ton TG, Fogg TT, Fong CT, John C, Li SX, Marshall JA, Peters K, Neal W, Pearson TA (2011). Knowledge, perception, and behaviors of relatives of people with premature heart disease: a systematic literature review. Circulation.

[16] World Health Organization. The WHO STEPwise approach to noncommunicable disease risk factor surveillance (STEPS). Geneva: World Health Organization. 2003. http://www.who.int/ncd_surveillance/en/steps_framework_dec03.pdf. 10.2105/AJPH.2015.302962 PMC4695948 26696288

[17] Jeemon P, Narayanan G, Kondal D, Kahol K, Bharadwaj A, Purty A, Negi P, Ladhani S, Sanghvi J, Singh K (2016). Task shifting of frontline community health workers for cardiovascular risk reduction: design and rationale of a cluster randomised controlled trial (DISHA study) in India. BMC Public Health.

[18] Lloyd-Jones DM, Hong Y, Labarthe D, Mozaffarian D, Appel LJ, Van Horn L, Greenlund K, Daniels S, Nichol G, Tomaselli GF (2010). Defining and setting national goals for cardiovascular health promotion and disease reduction: the American Heart Association’s strategic Impact Goal through 2020 and beyond. Circulation.

[19] D’Agostino RB, Grundy S, Sullivan LM, Wilson P, Group CHDRP (2001). Validation of the Framingham coronary heart disease prediction scores: results of a multiple ethnic groups investigation. JAMA.

[20] Mendis S, Lindholm LH, Mancia G, Whitworth J, Alderman M, Lim S, Heagerty T (2007). World Health Organization (WHO) and International Society of Hypertension (ISH) risk prediction charts: assessment of cardiovascular risk for prevention and control of cardiovascular disease in low and middle-income countries. J Hypertens.

[21] McGorrian C, Yusuf S, Islam S, Jung H, Rangarajan S, Avezum A, Prabhakaran D, Almahmeed W, Rumboldt Z, Budaj A (2011). Estimating modifiable coronary heart disease risk in multiple regions of the world: the INTERHEART Modifiable Risk Score. Eur Heart J.

[22] Batistatou E, Roberts C, Roberts S (2014). Sample size and power calculations for trials and quasiexperimental studies with clustering. The Stata Journal.

[23] Hanley JA, Negassa A, Edwardes MD, Forrester JE (2003). Statistical analysis of correlated data using generalized estimating equations: an orientation. Am J Epidemiol.

[24] Hernan MA, Brumback B, Robins JM (2000). Marginal structural models to estimate the causal effect of zidovudine on the survival of HIV-positive men. Epidemiology.

[25] Stone PW, Chapman RH, Sandberg EA, Liljas B, Neumann PJ (2000). Measuring costs in cost-utility analyses. Variations in the literature. Int J Technol Assess Health Care.

[26] Gilks W, Richardson S, Spiegelhalter D (1996). Markov Chain Monte Carlo in practice.

[27] Sachs J (2001). Macroeconomics and health: investing in health for economic development.

[28] Evans DB, Edejer TT, Adam T, Lim SS (2005). Methods to assess the costs and health effects of interventions for improving health in developing countries. BMJ.

[29] Shillcutt SD, Walker DG, Goodman CA, Mills AJ (2009). Cost effectiveness in low- and middle-income countries: a review of the debates surrounding decision rules. Pharmacoeconomics.

[30] Broderick C (1993). Understanding FAMILY PROCESS: Basics of Family Systems Theory.

[31] McCubbn HI, Patterson JM (1983). The family stress process: the double ABCX model of adjustment and adaptation. Marriage Fam Rev.

[32] Olson DH, Sprenkle DH, Russell CS (1979). Circumplex model of marital and family system: I. Cohesion and adaptability dimensions, family types, and clinical applications. Fam Process.

[33] Daivadanam M, Wahlstrom R, Thankappan KR, Ravindran TK (2015). Balancing expectations amidst limitations: the dynamics of food decision-making in rural Kerala. BMC Public Health.

[34] Gaziano T, Abrahams-Gessel S, Surka S, Sy S, Pandya A, Denman CA, Mendoza C, Puoane T, Levitt NS (2015). Cardiovascular disease screening by community health workers can be cost-effective in low-resource countries. Health Aff (Millwood).

[35] Uthman OA, Hartley L, Rees K, Taylor F, Ebrahim S, Clarke A (2015). Multiple risk factor interventions for primary prevention of cardiovascular disease in low- and middle-income countries. Cochrane Database Syst Rev.

